# Study on the outcome of patients with aseptic femoral head necrosis treated with percutaneous multiple small-diameter drilling core decompression: a retrospective cohort study based on magnetic resonance imaging and equivalent sphere model analysis

**DOI:** 10.1186/s13018-020-01786-4

**Published:** 2020-07-15

**Authors:** Yang Tan, Hangyuan He, Zihao Wan, Jun Qin, Yinxian Wen, Zhengqi Pan, Hua Wang, Liaobin Chen

**Affiliations:** Department of Joint Surgery and Sports Medicine, Zhongnan Hospital of Wuhan University, Wuhan, 430071 China

**Keywords:** Aseptic femoral head necrosis, Percutaneous multiple small-diameter drilling core decompression, Magnetic resonance imaging, Equivalent sphere model analysis

## Abstract

**Background:**

Aseptic necrosis of the femoral head (ANFH) has a high incidence in the community and causes substantial problems with health as well as economic and social stress. Core decompression is the most commonly used treatment for early ANFH. Although many studies have reported on the efficacy of femoral head core decompression surgery for ANFH, there are still some shortcomings in assessing the severity of femoral head necrosis, the location distribution, and changes in necrotic lesions before and after surgery. Magnetic resonance imaging (MRI) and equivalent sphere model analysis were used to further clarify the clinical efficacy of percutaneous multiple small-diameter drilling core decompression in patients with ANFH.

**Methods:**

From July 2013 to November 2016, 24 patients (32 cases of the hip joint) with ANFH who underwent percutaneous multiple small-diameter drilling core decompression were selected, and a retrospective analysis was conducted. MRI as well as VAS, OHS-C, and HHS scores were used to evaluate joint function in all patients before and 6, 12, and 24 months after the operation.

**Results:**

Twenty-four months after the operation, 10 hips were amputated. The survival rates of alcoholic femoral head necrosis (AFNH), idiopathic femoral head necrosis (IFHN), and steroid-induced femoral head necrosis (SIFHN) patients at 24 months were 100%, 85.7% (− 2 hips), and 0.0% (− 8 hips), respectively. The MRI and equivalent sphere analysis results revealed that the anterior superior medial quadrant was the area most prone to osteonecrosis, and the posterior superior medial quadrant was the area second most prone to necrosis. After the operation, the average percentage of the AFHN necrosis area in the total volume of the femoral head decreased from 14.5 to 10.3%, and the average percentage of the IFHN necrosis area decreased from 16.3 to 9.2%; however, the average percentage of the necrosis area for SIFHN increased from 30.4 to 33.1%.

**Conclusion:**

Percutaneous multiple small-diameter drilling core decompression significantly reduced the lesion volume for AFHN and IFHN, but the effect on SIFHN was not good.

## Background

Aseptic necrosis of the femoral head (ANFH), also known as nontraumatic femoral head necrosis, is a pathological process caused by impaired blood supply to the femoral head [1]. This process induces demineralization, trabecular thinning, and subsequent collapse of the joint surface with fracture of subchondral bone [2]. It can be classified as alcoholic femoral head necrosis (AFNH), steroid-induced femoral head necrosis (SIFHN), and idiopathic femoral head necrosis (IFHN) depending on the causes. ANFH has a high incidence in young and middle-aged populations and eventually leads to a severe loss of hip function [3]. In the USA, approximately 600,000 people are affected by ANFH [4]. The incidence of ANFH is also rising in Asian countries [5, 6]. The total population of individuals with ANFH in China over 15 years old is about 8.12 million [6]. This evidence suggests that ANFH has a high incidence in the community and causes substantial problems with health as well as economic and social stress.

Studies have shown that approximately 85% of patients have clinical symptoms, and 67% of patients without clinical symptoms experience a subsequent collapse of the femoral head; ultimately, these patients need to undergo artificial joint replacement [7]. To delay and reduce the probability of hip replacement in patients with ANFH, a series of interventions can be performed in patients with early femoral head necrosis, including biophysical therapy, drug intervention, bone flap transplantation with vascular pedicles, and core decompression of the femoral head [8]. Core decompression is the most commonly used treatment for early ANFH [9]. It improves local microvascular circulation by reducing the high pressure of the femoral head caused by tissue edema and promotes the healing of necrosis. Ultimately, core decompression relieves pain, improves joint function, and improves quality of life [10–12]. Although many studies have reported on the efficacy of femoral head core decompression surgery for ANFH, there are still some shortcomings in assessing the severity of femoral head necrosis, the location distribution, and changes in necrotic lesions before and after surgery.

The volume and location of femoral head necrosis are the main influencing factors associated with femoral head collapse [13]. However, the anatomical features of the femoral head limit our ability to accurately describe the differences between individuals with different types of femoral head necrosis. Magnetic resonance imaging (MRI) has a higher sensitivity and specificity for diagnosing early ANFH than X-ray and computed tomography (CT) examinations [14]. In addition, based on the definition of an equivalent sphere model for the femoral head [15], we can calculate the percentage of lesion volume in the equivalent sphere to represent the actual lesion volume across the femoral head [16]. Thus, we can assess the severity and prognosis of femoral head necrosis accurately and objectively.

The present study was characterized by a quantitative study of femoral head necrosis in ANFH patients treated with percutaneous multiple small-diameter drilling core decompression using MRI examination and equivalent sphere analysis. At the same time, the pain, function, and quality of life of ANFH patients were objectively described by various internationally recognized evaluation scales. Finally, a comprehensive assessment of the effect of percutaneous multiple small-diameter drilling core decompression on the prognosis of ANFH was conducted. This study is helpful for further clarifying the clinical efficacy of percutaneous multiple small-diameter drilling core decompression in patients with ANFH. It provides a theoretical basis for the treatment of ANFH by percutaneous multiple small-diameter drilling core decompression.

## Material and methods

### General information

**Fig. 1:**
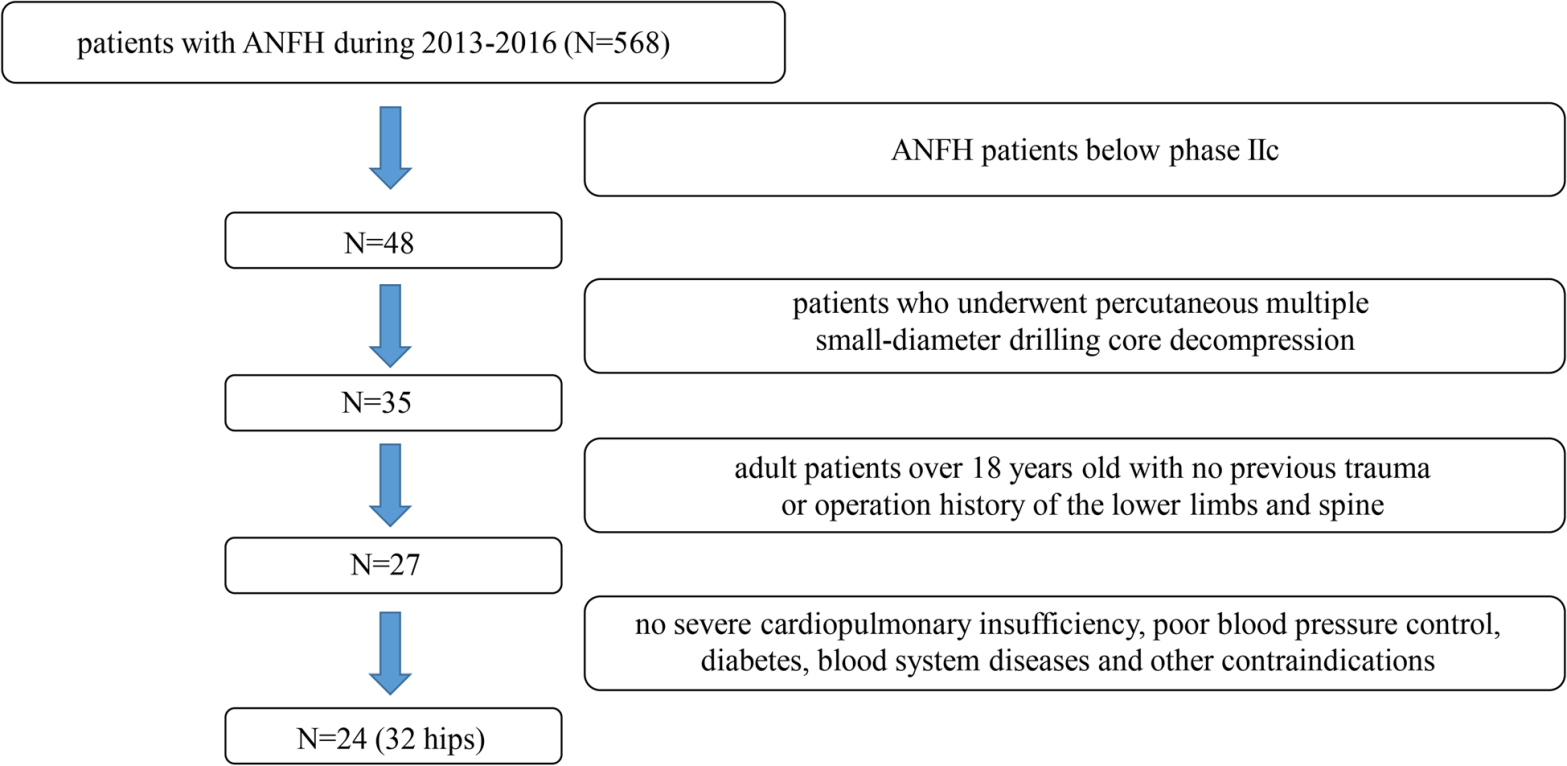
Flowchart of inclusion criteria. ANFH: aseptic necrosis of the femoral head

From July 2013 to November 2016, 24 patients (32 cases of the hip joint) with ANFH who underwent percutaneous multiple small-diameter drilling core decompression at the Zhongnan Hospital of Wuhan University were selected and subjected to retrospective analysis. All patients were examined with MRI and X-ray before admission, and the degree of necrosis of the femoral head was graded according to the method of the University of Pennsylvania. At the same time, the VAS score [17], OHS-C score [18], and HHS score [19] were used for preoperative evaluation. The inclusion process of the patients is shown in Fig. 1. The inclusion criteria were as follows: (a) patients with ANFH below stage IIc, (b) patients who voluntarily underwent core decompression surgery, (c) adult patients over 18 years old with no previous trauma or operation history of the lower limbs and spine, and (d) no contraindications such as severe cardiopulmonary insufficiency, poor control of blood pressure, diabetes, or blood system diseases.

### Operation method

**Fig. 2:**
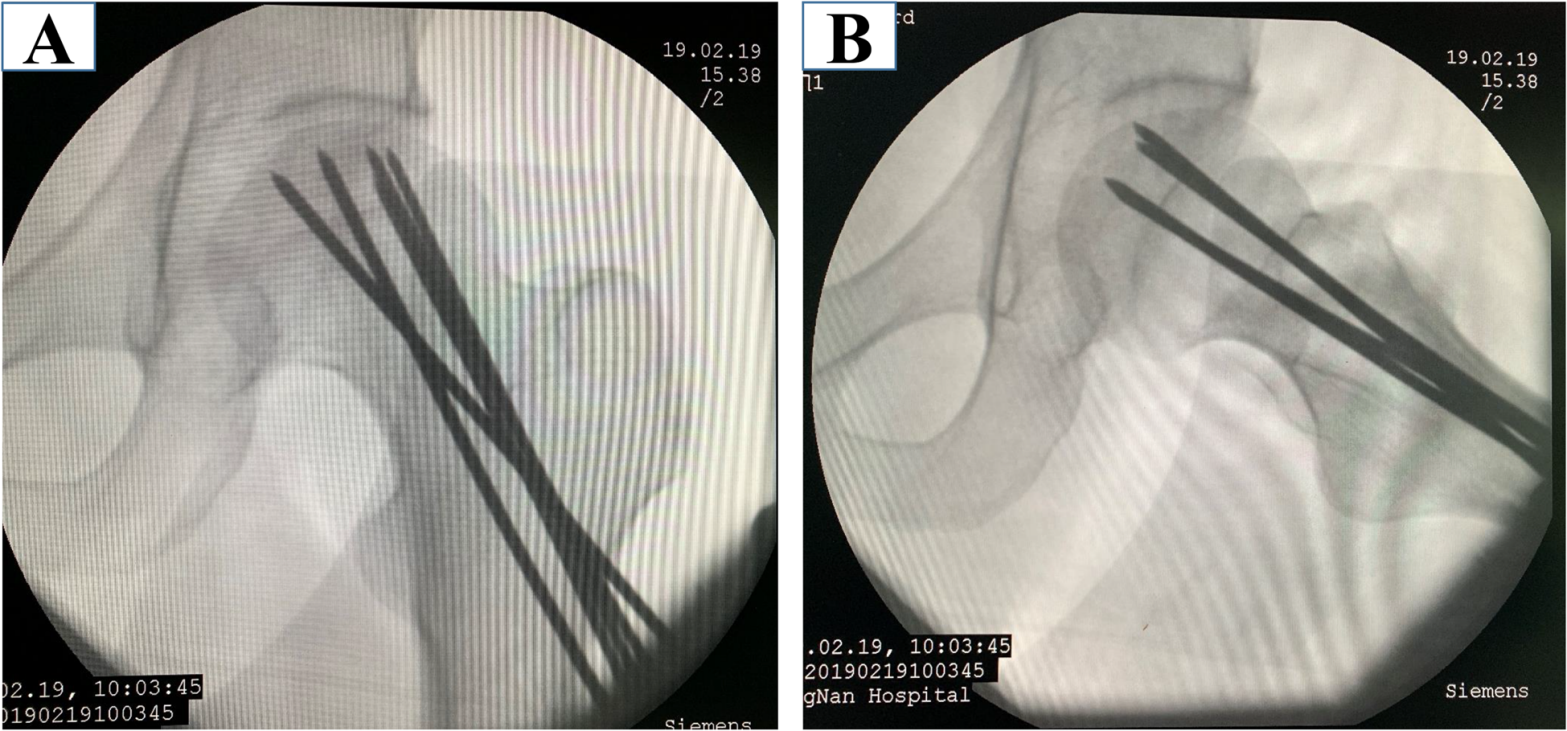
Three 2.5-mm-diameter Kirschner wires and two 3.0-mm-diameter Steinman pins were drilled into the femoral head

The patient was placed in a supine position after combined spinal-epidural anesthesia, which was convenient for intraoperative fluoroscopy. A 1% active iodine solution was used to disinfect the operation area, and then, a sterile dressing was used to cover the operation field. Three 2.5-mm-diameter Kirschner wires and two 3.0-mm-diameter Steinman pins were drilling through the femoral neck and femoral head under the trochanter to establish five decompression channels. Intraoperative fluoroscopy was used to ensure that the tip of the nail reached the necrotic area (Fig. 2).

### Postoperative rehabilitation

Patients were asked to perform a non-weight-bearing rehabilitation exercise in bed and allowed to walk with non-weight-bearing crutch for 12 weeks after surgery. Isometric leg contraction and ankle rotation training were carried out on the bed, following the principle of step-by-step strengthening. After 12 weeks, the patient began partial weight-bearing rehabilitation training with the help of a professional rehabilitation physician.

### Assessment instrument

VAS, OHS-C, and HHS scores were used to evaluate joint function within 1 week before the operation and at the 6, 12, and 24 months follow-up after the operation.

### Quantification of lesions based on the concept of MRI and equivalent sphere analysis

MRI and X-ray examinations were performed in all the subjects before the surgery and 6, 12, and 24 months after the operation. Cases of femoral head collapse and artificial joint replacement were recorded. Based on the concept of the equivalent sphere proposed by Malizos et al. [15], the equivalent sphere volume of the femoral head was larger than that of the corresponding anatomic entity. The equivalent sphere was divided into eight quadrants from the horizontal, coronal, and sagittal positions: anterior superior lateral (ASL), anterior superior medial (ASM), anterior inferior lateral (AIL), anterior inferior medial (AIM), posterior superior lateral (PSL), posterior superior medial (PSM), posterior inferior lateral (PIL), and posterior inferior medial (PIM). The volume percentage of lesions on the equivalent sphere was used to describe the volume ratio of the actual lesion size in the whole femoral head. Further, the severity of femoral head necrosis between different individuals was standardized and compared with each other. Two imaging specialists and one joint surgeon completed the measurement. Each person repeated the analysis twice, with an interval of at least 1 week each time. Controversial results were debated and measured by another senior joint surgeon. Finally, all the results were summarized, and the average value was calculated for analysis.

### Statistical methods

All data in this study were analyzed with SPSS 23.0 (IBM Corp., USA) software. In this study, the *t* test was used for statistical analysis. Statistical significance was designated at *P* < 0.05.

## Results

### General information about the research subjects

In this study, a total of 24 patients (including 32 hips) with ANFH were included, 18 males (24 hips) and 6 females (8 hips), with an average age of 47.1 ± 10.3 (20–62) years. Eight patients (10 hips) had AFNH, six patients (8 hips) had SIFHN, and ten patients (14 hips) had IFHN. According to the Pennsylvania stage of femoral head necrosis, there were 4 hips with stage Ia, 6 hips with stage Ib, 4 hips with stage Ic, 4 hips with stage IIa, 4 hips with stage IIb, and 10 hips with stage IIc disease. All patients were followed up for more than 6 months. The mean follow-up time was 25.9 ± 15.0 months (9.0–55.0 months) (Table 1).

**Table 1:** General information of the subjects included in the study

General information	Number
**Gender**	
Male	18 (24 hips)
Female	6 (8 hips)
**Average age (years)**	47.1 ± 10.3 (20–62)
**Pathogeny**	
AFHN	8 (10 hips)
SIFHN	6 (8 hips)
IFHN	10 (14 hips)
**Stage**	
Ia	4
Ib	6
Ic	4
IIa	4
IIb	4
IIc	10
**Follow-up time (months)**	25.9 ± 15.0 (9.0–55.0)

*AFHN* alcoholic femoral head necrosis, *SIFHN* steroid-induced femoral head necrosis, *IFHN* idiopathic femoral head necrosis

### Comparison of VAS, OHS-C, and HHS scores before and after the operation

The average VAS score was 2.4 ± 0.9 6 months after the core decompression operation, which was significantly lower than the score before the operation (Table 2, *P* < 0.01). At the end of the follow-up, the average VAS score was 3.1 ± 3.2, which was significantly lower than the score before core decompression (Table 2, *P* < 0.05).

**Table 2:** VAS scores before and after the operation

	Preoperative	6 months after the operation	End of follow-up
**Stage**			
Phase I	5.0 ± 0.8	2.0 ± 0.8**	0.4 ± 0.5**
Phase II	5.1 ± 0.7	2.8 ± 0.8**	5.1 ± 2.9
**Pathogeny**			
AFHN	4.8 ± 0.8	1.8 ± 0.8**	0.2 ± 0.4**
SIFHN	5.25 ± 0.9	3.25 ± 0.9**	7.25 ± 0.9**
IFHN	5.1 ± 0.7	2.4 ± 0.5**	2.7 ± 2.6*
**Total**	5.1 ± 0.8	2.4 ± 0.9**	3.1 ± 3.2*

*AFHN* alcoholic femoral head necrosis, *SIFHN* steroid-induced femoral head necrosis, *IFHN* idiopathic femoral head necrosis

*P < 0.05; **P < 0.01

The mean value of the OHS-C score of the hip joint at the 6-month follow-up was 35.6 ± 7.8, which was much higher than the score before the operation (Table 3, *P* < 0.01). At the end of the follow-up, the total average OHS-C score of the hip joint was 34.6 ± 13.7, which was significantly higher than the score before the operation (Table 3, *P* < 0.05).

**Table 3:** OHS-C scores before and after the operation

	Preoperative	6 months after the operation	End of follow-up
**Stage**			
Phase I	24.7 ± 1.9	41.9 ± 3.5**	45.6 ± 3.2**
Phase II	22.4 ± 2.2	30.8 ± 6.6*	26.0 ± 12.5
**Pathogeny**			
AFHN	24.2 ± 1.9	43.4 ± 2.8**	47.2 ± 1.8**
SIFHN	22.0 ± 2.6	27.3 ± 3.3*	17.0 ± 2.9**
IFHN	23.7 ± 2.4	34.8 ± 6.4*	35.6 ± 11.1*
**Total**	23.4 ± 2.3	35.6 ± 7.8**	34.6 ± 13.7*

*AFHN* alcoholic femoral head necrosis, *SIFHN* steroid-induced femoral head necrosis, *IFHN* idiopathic femoral head necrosis

*P < 0.05; **P < 0.01

At the 6-month follow-up, the average HHS score of the hip joint was 78.2 ± 9.1, which was much higher than the score before the operation (Table 4, *P* < 0.01). At the end of the follow-up, the average HHS score of the hip joint was 78.7 ± 17.4, which was higher than the score before the operation (Table 4, *P* < 0.05).

**Table 4:** HHS scores before and after the operation

	Preoperative	6 months after the operation	End of follow-up
**Stage**			
Phase I	76.9 ± 4.9	86.9 ± 2.5**	93.2 ± 3.3**
Phase II	66.5 ± 5.8	71.4 ± 5.4**	67.4 ± 15.3
**Pathogeny**			
AFHN	78.3 ± 5.3	87.8 ± 2.4**	94.0 ± 3.2**
SIFHN	61.9 ± 5.3	66.9 ± 2.7*	55.0 ± 3.3*
IFHN	71.1 ± 3.0	77.8 ± 5.9*	81.2 ± 12.9*
**Total**	71.1 ± 7.5	78.2 ± 9.1**	78.7 ± 17.4*

*AFHN* alcoholic femoral head necrosis, *SIFHN* steroid-induced femoral head necrosis, *IFHN* idiopathic femoral head necrosis

*P < 0.05; **P < 0.01

When analyzed according to pathogeny, the results suggested that percutaneous multiple small-diameter drilling core decompression significantly improved VAS, OHS-C, and HHS scores in patients with AFHN or IFHN. However, as shown in Tables 2, 3, and 4, core decompression only helped for the VAS, OHS-C, and HHS scores of patients with SIFHN within 6 months and did not improve hip pain or function in the middle period (more than 12 months).

### Survival analysis of the hips

**Fig. 3:**
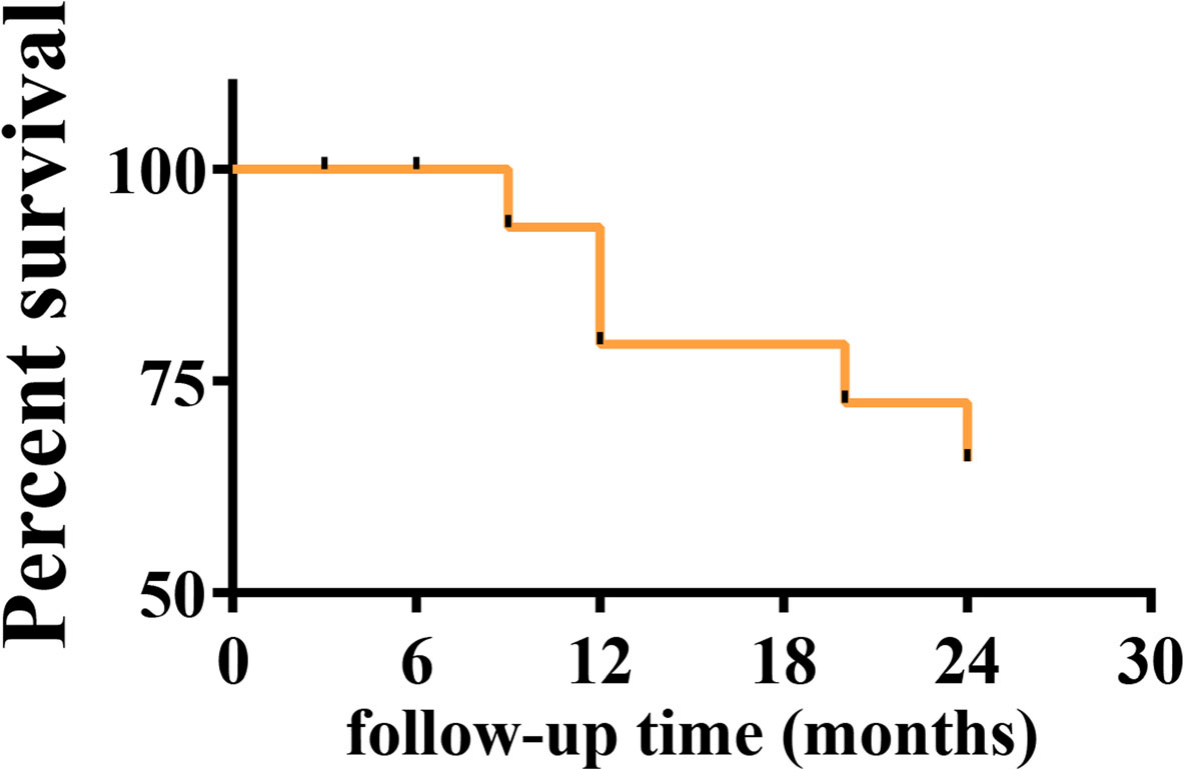
Overall survival curve of the hip joint after the operation

**Fig. 4:**
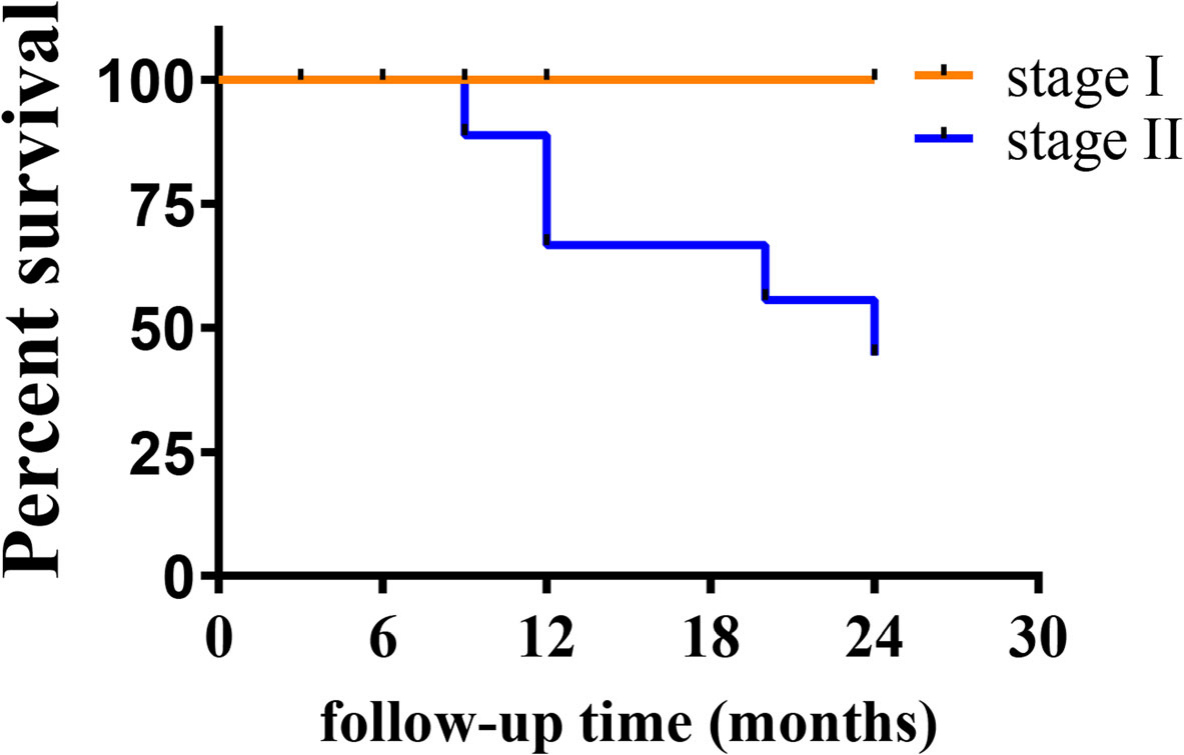
Postoperative hip survival curve of the femoral head at different stages

**Fig. 5:**
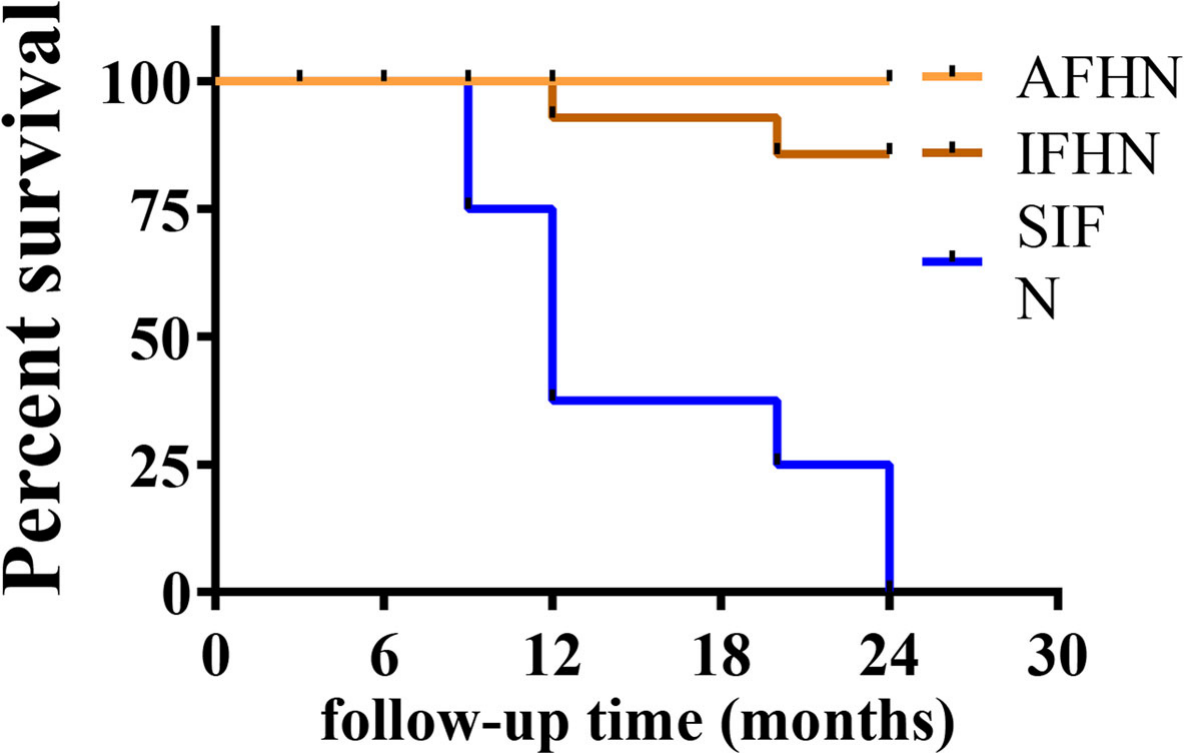
Postoperative hip survival curve of aseptic necrosis of the femoral head with different pathogeny. AFHN: alcoholic femoral head necrosis; SIFHN: steroid-induced femoral head necrosis; IFHN: idiopathic femoral head necrosis

Twenty-four months after core decompression, 10 hips were amputated (femoral head collapse), and all of them had stage II disease before the operation. Among them, 2 hips collapsed at 9th month, 4 hips collapsed at 12th month, 2 hips collapsed at 20th month, and 2 hips collapsed at 24th month after the surgery. The overall survival rates at 12 and 24 months in this study were 81.3% and 68.6%, respectively (Fig. 3). The 24-month survival rate for stage I ANFH was 100%. The survival rates for stage II ANFH at 12 and 24 months were 66.7% and 44.4%, respectively (Fig. 4). The 24-month survival rate of AFHN was 100%. The survival rates of IFHN at 12 and 24 months were 92.6% (− 1 hip) and 85.7% (− 2 hips), respectively. The survival rates of SIFHN at 12 and 24 months were 37.5% (− 5 hips) and 0.0% (− 8 hips), respectively (Fig. 5).

### Volume and distribution of femoral head necrosis area before the operation

**Fig. 6:**
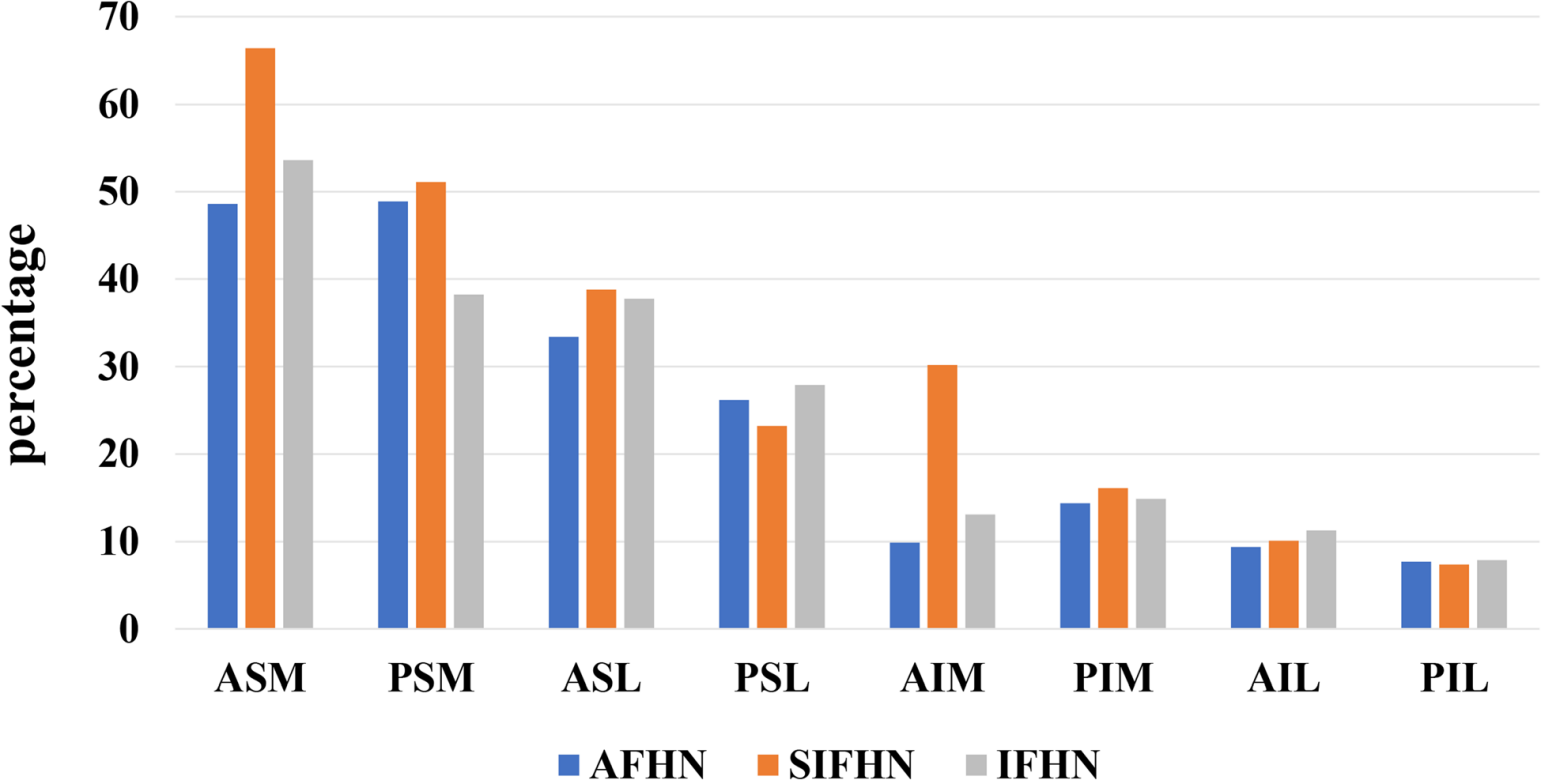
Distribution of the femoral head necrosis area before the operation. ASM: anterior superior medial; PSM: posterior superior medial; ASL: anterior superior lateral; PSL: posterior superior lateral; AIM: anterior inferior medial; PIM: posterior inferior medial; AIL: anterior inferior lateral; PIL: posterior inferior lateral; AFHN: alcoholic femoral head necrosis; SIFHN: steroid-induced femoral head necrosis; IFHN: idiopathic femoral head necrosis

The preoperative evaluation showed that the average volume in focus in AFHN was 14.1 ± 4.3 cm^3^, accounting for 24.8% ± 7.2% of the total volume of the femoral head. The average volume of the necrosis area for SIFHN was 16.5 ± 2.4 cm^3^, accounting for 30.4% ± 5.4% of the total volume of the femoral head. The average volume of the necrosis area for IFHN was 14.7 ± 6.0 cm^3^, accounting for 25.6% ± 8.7% of the total volume of the femoral head (Table 5). The average percentage of the total necrosis area in the volume of the femoral head in the study was 26.5% ± 7.5% (Table 5). Among these areas, the ASM quadrant was the most prone area for osteonecrosis. The average percentage of the volume of osteonecrosis area in the total volume of this quadrant was 55.2% ± 18.6% (Table 5/Fig. 6). The smallest affected area was the PIL quadrant, and the mean volume percentages of the dead and damaged objects were 7.7% ± 3.1% (Table 5/Fig. 6).

**Table 5:** Volume and distribution of the femoral head necrosis area before the operation

Percentage	AFHN	SIFHN	IFHN	All
**ASM**	48.6 ± 12.3	66.4 ± 12.2	53.6 ± 23.9	55.2 ± 18.6
**PSM**	48.9 ± 14.5	51.1 ± 22.9	38.2 ± 13.5	44.7 ± 16.4
**ASL**	33.4 ± 15.9	38.8 ± 17.1	37.8 ± 16.2	36.7 ± 15.4
**PSL**	26.2 ± 9.5	23.2 ± 10.8	27.9 ± 13.8	26.2 ± 11.3
**AIM**	9.9 ± 4.1	30.2 ± 11.3	13.1 ± 7.1	16.3 ± 10.9
**PIM**	14.4 ± 4.8	16.1 ± 4.0	14.9 ± 8.9	15.1 ± 6.4
**AIL**	9.4 ± 6.9	10.1 ± 4.6	11.3 ± 8.7	10.4 ± 6.9
**PIL**	7.7 ± 3.4	7.4 ± 2.6	7.9 ± 3.5	7.7 ± 3.1
Mean	24.8 ± 7.2	30.4 ± 5.4	25.6 ± 8.7	26.5 ± 7.5

*ASM* anterior superior medial, *PSM* posterior superior medial, *ASL* anterior superior lateral, *PSL* posterior superior lateral, *AIM* anterior inferior medial, *PIM* posterior inferior medial, *AIL* anterior inferior lateral, *PIL* posterior inferior lateral, *AFHN* alcoholic femoral head necrosis, *SIFHN* steroid-induced femoral head necrosis, *IFHN* idiopathic femoral head necrosis

### Volume and distribution of the femoral head necrosis area after the operation

**Fig. 7:**
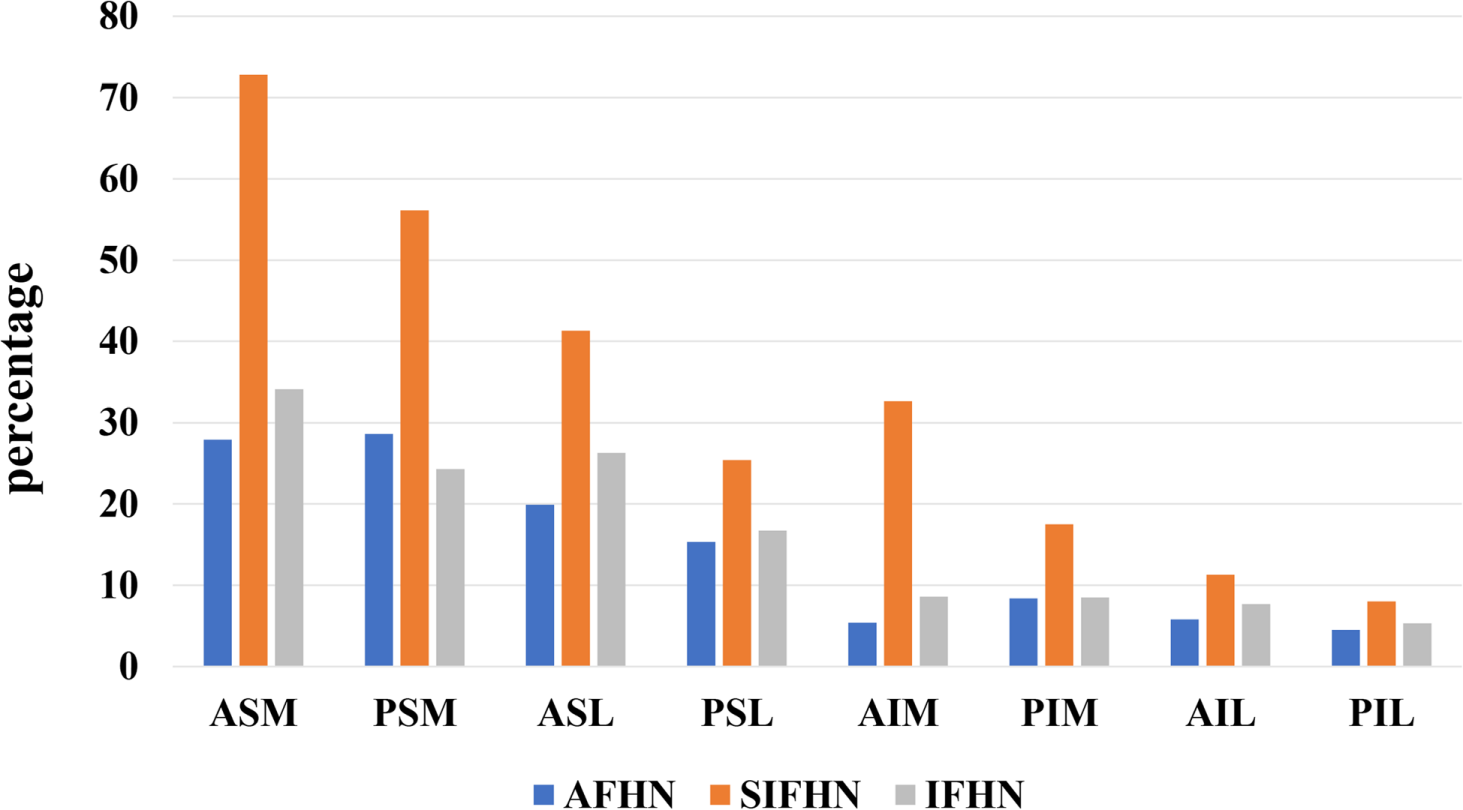
Volume and distribution of the femoral head necrosis area at the 6th month after the operation. Anterior superior medial (ASM), posterior superior medial (PSM), anterior superior lateral (ASL), posterior superior lateral (PSL), anterior inferior medial (AIM), posterior inferior medial (PIM), anterior inferior lateral (AIL), posterior inferior lateral (PIL). AFHN: alcoholic femoral head necrosis; SIFHN: steroid-induced femoral head necrosis; IFHN: idiopathic femoral head necrosis

Six months after core decompression, the average percentage of the total necrosis volume of the femoral head was 19.9% ± 10.1%, which was 6.5% ± 7.9% lower than the average before core decompression (Table 6). The most significant reduction in necrosis area volume was seen in the ASM quadrant. In the ASM quadrant, the average percentage of the necrosis area volume in the total volume of this quadrant was 41.8% ± 23.3%, which was 13.4% ± 17.4% lower than the average before the operation (Table 6/Fig. 7). The average percentage of the AFHN necrosis area in the total volume of the femoral head was 14.5% ± 6.6%, which was 10.3% ± 0.9% less than the average before the operation; the average percentage of the IFHN necrosis area was 16.3% ± 7.5%, which was 9.2% ± 8.7% less than the average before the operation. However, the mean volume percentage of the necrosis area for SIFHN was 33.1% ± 5.1%, which increased by 2.7% ± 2.6% compared with the average before surgery. The results of the subgroup analysis showed that the volume of the necrosis area in the ASM quadrant for SIFHN increased significantly (6.5% ± 6.6%) (Table 6/Fig. 7).

**Table 6:** Volume and distribution of the femoral head necrosis area at 6th month after the operation

Percentage	AFHN	SIFHN	IFHN	All	△*V* = (*V*_pre_ − *V*_post_)
**ASM**	27.9 ± 10.4	72.8 ± 15.9	34.1 ± 16.7	41.8 ± 23.3	13.4 ± 17.4
**PSM**	28.6 ± 13.2	56.1 ± 26.7	24.3 ± 9.6	33.6 ± 20.2	11.2 ± 13.4
**ASL**	19.9 ± 11.7	41.3 ± 14.6	26.3 ± 18.6	28.1 ± 16.9	8.6 ± 10.5
**PSL**	15.3 ± 7.8	25.4 ± 12.9	16.7 ± 6.7	18.4 ± 8.9	7.8 ± 9.4
**AIM**	5.4 ± 2.5	32.6 ± 10.8	8.6 ± 6.2	13.6 ± 13.5	2.7 ± 4.9
**PIM**	8.4 ± 4.2	17.5 ± 3.5	8.5 ± 4.4	10.7 ± 5.6	4.3 ± 5.1
**AIL**	5.8 ± 4.9	11.3 ± 5.9	7.7 ± 6.6	8.0 ± 5.9	2.4 ± 3.8
**PIL**	4.5 ± 2.7	8.0 ± 2.5	5.3 ± 3.2	5.8 ± 3.0	1.9 ± 2.5
**Mean**	14.5 ± 6.6	33.1 ± 5.1	16.3 ± 7.5	19.9 ± 10.1	6.5 ± 7.9
△*V* = (*V*_pre_ − V_post_)	10.3 ± 0.9	− (2.7 ± 2.6)	9.2 ± 8.7	6.5 ± 7.9	−

*ASM* anterior superior medial, *PSM* posterior superior medial, *ASL* anterior superior lateral, *PSL* posterior superior lateral, *AIM* anterior inferior medial, *PIM* posterior inferior medial, *AIL* anterior inferior lateral, *PIL* posterior inferior lateral, *AFHN* alcoholic femoral head necrosis, *SIFHN* steroid-induced femoral head necrosis, *IFHN* idiopathic femoral head necrosis

### Clinical information of patients with amputation

In order to further explore the factors affecting the efficacy of percutaneous multiple small-diameter drilling core decompression, we summarized the clinical characteristics of the patients with tail amputation events in this study. As shown in Table 7, a total of 10 tail amputations occurred in this study, among which 8 were SIFN and 2 were IFHN, suggesting that the effect of decompression of SIFN with percutaneous fine-needle core was not good. VAS, OHS-C, and HHS in patients with tail amputation events were not significantly improved compared with those before surgery. In terms of the distribution of collapse area, 10 cases of hip joints collapsed in ASM, 9 cases collapsed in PSM, 8 cases collapsed in ASL, and 5 cases collapsed in PSL. The collapse area was mainly concentrated in the weight-bearing area. It was suggested that percutaneous multiple small-diameter drilling core decompression was not effective for SIFN, and the load-bearing area was the main area when the collapse occurred.

**Table 7:** Clinical information of patients with amputation

Diagnosis	Phase	Post-operative time (months)	Scores						Distribution of collapse area			
			VAS		OHS-C		HHS					
			Preoperative	Final	Preoperative	Final	Preoperative	Final				
SIFN	IIc	9	6	8	20	17	52	44	ASM	PSM	ASL	PSL
SIFN	IIc	9	6	9	18	15	58	46	ASM	PSM	ASL	PSL
SIFN	IIc	12	5	7	20	16	59	51	ASM	PSM		PSL
SIFN	IIc	12	6	8	19	15	59	53	ASM		ASL	PSL
SIFN	IIb	12	7	9	23	19	62	42	ASM	PSM	ASL	
SIFN	IIa	20	5	8	24	18	59	43	ASM	PSM	ASL	
SIFN	IIc	24	4	8	25	14	58	51	ASM	PSM	ASL	
SIFN	IIb	24	7	10	27	16	64	56	ASM	PSM		PSL
IFHN	IIb	12	4	7	30	19	66	58	ASM	PSM	ASL	
IFHN	IIc	20	5	8	29	20	69	59	ASM	PSM	ASL	

*SIFHN* steroid-induced femoral head necrosis, *IFHN* idiopathic femoral head necrosis, *ASM* anterior superior medial, *PSM* posterior superior medial, *ASL* anterior superior lateral, *PSL* posterior superior lateral

## Discussion

Femoral head core decompression is the most commonly used surgery for the treatment of early ANFH. It reduces the internal pressure of the femoral head caused by tissue edema and promotes bone regeneration, healing, and remodeling, and thus reduces pain, improves joint function, and improves quality of life [10–12]. Studies have proven that the percutaneous multiple small-diameter drilling core decompression can relieve the pain of the hip joint in patients and delay the progress of femoral head necrosis [20–22]. According to Mont et al., 63.5% of reviewed hips achieved a well clinical result after core decompression. Stage predicted outcome, with a survival rate of 84% of hips with stage I ANFH and 65% of hips with stage II ANFH [23]. According to the results of Song et al., the survival rate of stage I ANFH and stage II ANFH was 79% and 77%, respectively, after multiple drilling core decompression for 5 years [24]. In this study, after the operation, the VAS scores decreased, and the HHS and OHS-C scores increased in all patients with AFHN or IFHN. These results suggested that percutaneous multiple small-diameter drilling core decompression significantly improved the pain, quality of life, and hip function of patients with AFHN or IFHN. However, for patients with SIFHN, percutaneous multiple small-diameter drilling core decompression improved the VAS, HHS, and OHS-C scores in a short period, but had no effect on the long-term VAS, HHS, and OHS-C scores. In general, percutaneous multiple small-diameter drilling core decompression significantly improved the short-term and long-term joint health and quality of life of patients with AFHN or IFHN. However, for SIFHN, percutaneous multiple small-diameter drilling core decompression only improved the local symptoms of the joint in the short term and had limited long-term effects. In addition, the survival rate of stage I ANFH was 100% and stage II ANFH was 44% at 2 years after the percutaneous multiple small-diameter drilling core decompression in this study. The results were similar to previous reports.

In current clinical studies, early treatment of ANFH emphasizes comprehensive treatment. The conventional methods of treatment include core decompression combined with weight-bearing restriction and drug [25, 26]. Most studies have shown that after core decompression surgery, patients need to undergo a 3–6-month weight-bearing restriction to achieve better outcomes [27–29]. The post-operative weight-bearing restriction is helpful for the recovery of blood circulation in the femoral head of the affected limb. Moreover, it can promote the regeneration and remodeling of the internal bone of the femoral head, delay the further necrosis of the femoral head, and ultimately improve the success rate of core decompression surgery [26]. However, it has been reported that for non-surgical patients, the survival rate of the femoral head could not be improved simply by weight-bearing restriction, and the progression of femoral head necrosis was not significantly delayed [25, 30]. A retrospective study by Mont et al. showed that more than 74% of patients with femoral head necrosis were further aggravated after 34 months of weight-bearing restriction [25]. In addition, animal experiments showed that there was no significant difference in the progression of steroid-induced femoral head necrosis in the rat model between the control group and the weight-bearing-restricted group [31]. In this study, all patients underwent core decompression. After 6 months of weight-bearing restriction, the hip joint score was significantly improved and femoral head necrosis progression was significantly delayed. Therefore, we speculated that the improvement in various clinical scores of patients after core decompression surgery was mainly caused by the surgery, rather than the long-term non-weight bearing.

The volume and location of femoral head necrosis are the main factors related to the collapse of the femoral head. However, the anatomical characteristics of the femoral head often limit researchers in describing the differences between different individuals. MRI demonstrates the volume and location distribution of femoral head necrosis foci in three dimensions, which can more accurately evaluate the severity and prognosis of femoral head necrosis. Based on the definition of an equivalent sphere model for the femoral head, MRI can help calculate the volume percentage of femoral head tissue affected by avascular necrosis in different regions and compare the differences between different individuals [15]. The prognosis of ANFH is related to the volume and location distribution of femoral head necrosis lesions [32–34]. ANFH patients with micronecrotic lesions far from the articular surface may have a good prognosis even without additional therapeutic intervention. However, the prognosis of ANFH patients with large necrotic lesions involving subchondral bone in the weight-bearing region is poor whether treated or not [32, 35]. Therefore, it is of great significance for the treatment of ANFH to accurately describe the lesion distribution in the femoral head with three-dimensions by using the equivalent sphere-volume method. In this study, we found that the focus of ischemic necrosis in ANFH patients was mainly located in the ASM quadrant, which was basically consistent with previous studies. These findings also suggest that the direction of the decompression guidewire should be as close as possible to the anterior-upper part of the femoral head when performing femoral head core decompression.

In ANFH patients without early intervention, deformation and collapse of the femoral head will occur within 2 years of onset as the necrosis volume of the femoral head expands. These patients develop osteoarthritis of the hip and eventually require an artificial joint replacement. Therefore, the volume of the necrotic area of the femoral head is an important indicator of the degree of necrosis of the femoral head [36]. In this study, compared with the preoperative volume, the volume of the total necrotic lesion of the hip decreased 6 months after decompression surgery. Among these volumes, the necrotic volume of the ASM region in the main load area was significantly reduced, while the necrotic volumes of other areas were decreased to varying degrees. These results suggest that percutaneous multiple small-diameter drilling core decompression reduces the lesion volume of patients with femoral head necrosis, thus delaying the progress of femoral head necrosis. However, all 6 patients (8 hips) with SIFHN included in this study were found to have a larger volume of osteonecrosis of the femoral head on MRI 6 months after surgery compared with that of before surgery. All 6 patients (8 hips) with SIFHN had undergone artificial joint replacement at the end of the follow-up. SIFHN is associated with angiogenic dysfunction, decreased bone repair ability, and inhibited BMP-2 receptor expression [37–39]. Although decompression with a femoral head core drill releases intramedullary pressure and improves intramedullary microcirculation, it cannot improve bone cell metabolism and bone regeneration function for SIFHN. Therefore, the effect of percutaneous multiple small-diameter drilling core decompression on SIFHN is limited.

## Limitations of this study

This study is a retrospective study, and the quality of evidence is lower than that of prospective studies and randomized controlled trials. Further, the number of cases included in the study was small. The lack of stage I SIFHN cases may be a factor affecting the prognosis of SIFHN. Further expansion of the sample size and a long-term follow-up are needed in the future.

## Conclusion

The equivalent sphere method based on MRI can accurately describe the location distribution of ANFH lesions, as well as the volume change of the necrotic area before and after the operation. The short-term (within 6 months) and long-term (more than 12 months) VAS, HHS, and OHS-C scores after surgery for AFHN and IFHN were significantly improved by percutaneous multiple small-diameter drilling core decompression, while the effect of SIFHN on long-term hip pain relief and functional improvement was limited. Percutaneous multiple small-diameter drilling core decompression significantly reduced the lesion volume for AFHN and IFHN, but the effect on SIFHN was not good.

## Data Availability

All the data of the manuscript are presented in the paper.

## Abbreviations

ANFH: Aseptic necrosis of the femoral head
MRI: Magnetic resonance imaging
AFHN: Alcoholic femoral head necrosis
IFHN: Idiopathic femoral head necrosis
SIFHN: Steroid-induced femoral head necrosis
CT: Computed tomography
ASL: Anterior superior lateral
ASM: Anterior superior medial
AIL: Anterior inferior lateral
AIM: Anterior inferior medial
PSL: Posterior superior lateral
PSM: Posterior superior medial
PIL: Posterior inferior lateral
PIM: Posterior inferior medial
